# Neurocognitive disparities: investigating ethnicity and mental health in rural aging adults

**DOI:** 10.18632/aging.206166

**Published:** 2024-11-27

**Authors:** Carol Fadalla, Jonathan Singer, Peter Rerick, Lauren Elliott, Elisabeth McLean, Sydnie Schneider, Lauren Chrzanowski, Veronica Molinar-Lopez, Volker Neugebauer

**Affiliations:** 1 Department of Psychological Science, Texas Tech University, Lubbock, TX 79410, USA; 2 Department of Psychology, University of Central Oklahoma, Edmond, OK 73034, USA; 3 Garrison Institute on Aging, Texas Tech University Health Sciences Center, Lubbock, TX 79430, USA; 4 Department of Pharmacology and Neuroscience, School of Medicine, Texas Tech University Health Sciences Center, Lubbock, TX 79430, USA

**Keywords:** rural disparities, aging adults, neurocognitive functioning, Hispanic individuals, depression, anxiety

## Abstract

Objectives: We explored whether depression and anxiety moderated the association of ethnicity and neurocognitive functioning among a sample of Hispanic and non-Hispanic White rural aging adults.

Method: 1,462 rural dwelling adults (*M*age = 59.4 years, *SDa*ge = 12.12) were included in the analysis for this study.

Results: MANCOVAs revealed a significant (*ps* < .001) multivariate effect of ethnicity on all five indices of neurocognitive functioning when controlling for anxiety and sociodemographic variables (*V* = .20, *F*(5,1,310) = 64.69) and depression and sociodemographic variables in the second model (*V* = .20, *F*(5,1310) = 65.80, *p* < .001). There was also a multivariate effect of anxiety (*V* = .02, *F*(5,1310) = 4.57, *p* < .001) and depression (*V* = .04, *F*(5, 1310) = 11.38, *p* < .001) on neurocognitive functioning when controlling for sociodemographic variables and ethnicity.

Conclusion: Findings revealed that Hispanic rural aging adults scored lower on neurocognitive functioning compared to non-Hispanic White rural aging adults, irrespective of depression or anxiety. Depression and anxiety contributed to lower scores on neurocognitive functioning—yet this finding was not as robust. Culturally tailored interventions targeting risk factors for neurocognitive impairment in Hispanic rural aging adults are imperative to mitigate neurocognitive disparities. One possible reason for differences in neurocognitive functioning between Hispanic individuals and non-Hispanic individuals is stress as ethnic health disparities have been found to be shaped by a diverse range of lifetime stressors that are disproportionally exacerbated for ethnic minorities.

## INTRODUCTION

By 2050, the number of aging adults (i.e., 65 and older) with Alzheimer’s Disease and Related dementia (ADRD) is forecasted to reach 13.89 million [1]. The increase in neurocognitive disorders is likely to disproportionately impact rural communities in the United States, where aging adults comprise 17% of the rural community as opposed to 13.8% in urban areas [2, 3]. Among rural-dwelling aging adults, the Hispanic population represents the largest share of the rural minority population (4.1 million) [4]. Alarmingly, Hispanic individuals face an elevated risk of neurocognitive disorders compared to non-Hispanic White populations, with studies indicating up to a 1.5 times higher likelihood of meeting the dementia diagnostic criteria [5].

Recent research suggests that Hispanic rural aging adults have lower levels of neurocognitive functioning than non-Hispanic rural aging adults [6–8]. The term “Hispanic” is an ethnicity that encapsulates individuals from diverse backgrounds such as Cuban, Dominican, Mexican, Puerto Rican, South or Central American, or other Spanish cultures regardless of race [9], whereas, “non-Hispanic” is any group that does not share a Spanish origin. Studies also suggest that Hispanic aging adults are more likely to be diagnosed with advanced stages of neurocognitive disorders at younger ages [7]. This consistent trend across studies [6, 10–14] implies that Hispanic rural aging adults might be uniquely at risk of lower neurocognitive functioning compared to other ethnic populations. Despite consistent evidence suggesting lower levels of neurocognitive functioning among Hispanic rural aging adults, a significant gap remains. Few studies have identified moderators for the association between Hispanic ethnicity to the risk of lower neurocognitive functioning.

While the underlying factors contributing to these disparities may be multifaceted, emerging evidence suggests that mood disorders, particularly depression and anxiety, may play a crucial role in moderating this relationship. Examining the role of mood disorders (i.e., depression and anxiety) and ethnicity could offer vital insights into the observed disparities in neurocognitive functioning among Hispanic rural aging adults. Particularly, the investigation of mood disorders may provide a potentially modifiable intervention target to attenuate these risks. We adopt the integrative biopsychosocial model as a framework [15] to explore the potential contribution of mood disorders in perpetuating ethnic disparities in neurocognitive functioning between Hispanic and non-Hispanic White rural aging adults.

### Lifespan biopsychosocial model of cumulative psychological vulnerability and minority health

The lifespan biopsychosocial model of cumulative psychological vulnerability and minority health [15, 16] provides insight into the ethnicity, mental health, and neurocognitive relationship. Under this framework, ethnic health disparities are shaped by a diverse range of lifetime stressors that are disproportionally exacerbated for ethnic minorities [15]. These lifetime stressors can lead to adverse health outcomes through a series of “reciprocal, recursive and synergistic interaction(s) among the major factors in the model over the life course (15(p16)).” Therefore, according to Myers’ (15(p16)) framework, these “race/ethnicity-related stressors not only make independent contributions to greater stress burden (additive effect), but may also exacerbate the impact of other life stressors (synergistic effect).” Indeed, literature has demonstrated that Hispanic rural aging adults are more likely to experience a variety of stressors that may either moderate, mediate, or independently contribute to their cumulative psychological vulnerability. These include greater social and geographical isolation [17, 18], lower educational attainment (e.g., 34.6% reported not completing high school compared to 10.4% of non-Hispanic White rural adults) [19], and heightened poverty levels compared to non-Hispanic White rural aging adults [19].

According to this model, unequivocal exposure to lifetime stressors for ethnic minorities may increase the likelihood of developing cognitive-emotional conditions (i.e., mood disorders) and increase the cumulative burden of morbidity and mortality over the life course [15, 16]. Although the original focus of this model was on detrimental biological disease outcomes (e.g., cardiovascular risk) [15], this model has been extended to psychosocial outcomes [8]. Further, studies have found that Hispanic aging adults are at increased risk for late-life depression (LLD) and late-life anxiety (LLA), which additionally supports this theory in the context of psychosocial factors. Therefore, this study investigates the synergistic effect, the potential moderation, of depression/anxiety and ethnicity on neurocognitive functioning. It should be noted that there are other factors that should be investigated that are in line with this theory that could play a role, inlcuding alcohol use, exersise, nutrion, and socialization.

### Late-life depression in aging Hispanic adults

LLD, characterized by persistent changes in mood, lack of pleasure, and somatic symptoms [20], significantly impacts the well-being of aging adults [21]. Various studies have found that Hispanic aging adults endorse higher severity of LLD and greater associated LLD burden [12, 22–27]. For example, Hispanic older adults were 14% more likely to endorse depression symptoms than non-Hispanic older adults and less likely to have reported being diagnosed or treated with depression [27]. These findings underscore the heightened prevalence and severity of LLD in Hispanic aging adults, which poses a considerable risk to their well-being. The rise in LLD is particularly problematic as LLD has been linked to a 50% increased risk of neurocognitive impairment [28].

### Late-life anxiety in aging Hispanic adults

LLA is characterized as a spectrum of excessive worry and fear that has been identified as a potential prelude to or sign of ADRD (i.e., particularly symptoms of agitation) [29]. While research on LLA among Hispanic aging adults is less extensive compared to LLD [25], research indicates Hispanics are 1.6 times more likely to report anxiety than other ethnic groups [25]. Additionally, the lifetime prevalence of anxiety is 18.8% more prevalent in Hispanic aging adults than non-Hispanic White aging adults [25]. The increased risk of LLA in Hispanic aging adults is a concern as LLA has been identified as a potential risk factor for neurocognitive impairment in aging adults, particularly affecting executive functioning [30]. These results are troubling given that Hispanic rural aging adults have a higher risk of neurocognitive impairment [6–8] and suggest that anxiety symptoms may further hasten neurocognitive impairments in high-risk populations.

### Mood disorders and neurocognitive functioning in Hispanic aging adults

Research on the relationship between mood disorders and neurocognitive functioning in Hispanic aging adults remains an underexplored area of investigation. However, available studies have consistently demonstrated that mood disorders are associated with lower neurocognitive functioning in Hispanic aging adults and ethnic disparities in neurocognitive functioning may be more pronounced among those with mood disorders [6–8, 10, 13, 31, 32]. In a cross-ethnic sample of persons with AD and mild cognitive impairment, depression and anxiety were more frequently endorsed in Hispanic persons than in non-Hispanic White persons [32]. Depression has been associated with an increased risk for mild cognitive impairment in Hispanic aging adults, whereas this association was not demonstrated with non-Hispanic Whites [31]. Camacho and colleagues [10] also found an association between anxiety and depression and delayed memory deficits in Hispanic aging adults. On the contrary, Hispanic aging adults with lower anxiety and depression were less likely to exhibit delayed memory deficits. Similarly, another cross-sectional study underscored depression as a contributing factor to diminished cognitive scores across neurocognitive assessments for Hispanic aging adults [13]. There was also a notable difference in neurocognitive scores between Hispanic subgroups, specifically Mexican Americans and Puerto Ricans. Notably, Mexican Americans, with lower depression scores, exhibited higher neurocognitive functioning. In contrast, Puerto Ricans, with higher depression scores, displayed poorer neurocognitive performance.

The discrepancy in neurocognitive scores influenced by depression implies depression may potentially moderate the relationship between neurocognitive functioning and ethnicity in Hispanic aging adults. Indeed, prior research has linked higher depression scores with diminished neurocognitive functioning in Mexican American rural aging adults [6, 7], suggesting that depression scores within Hispanic subgroups may relate to differences in neurocognitive scores. In addition, different facets of depression (such as apathy, dysphoria, cognitive impairment, and meaninglessness) have also been associated with lower overall neurocognitive functioning in Hispanic rural aging adults [6]. For instance, while dysphoria primarily affected delayed memory in non-Hispanic rural aging adults, a greater scope of depressive domains (i.e., dysphoria, meaninglessness, and cognitive impairment) predicted both immediate and delayed memory, visuospatial/visuoconstructive memory, and language scores for Hispanic rural aging adults [6]. Therefore, despite the limited scope of literature on this topic, preliminary evidence suggest that a wider range of depression and anxiety symptoms may be associated with lower levels of neurocognitive functioning among Hispanic rural aging adults, in comparison to non-Hispanic White rural aging adults.

### Research overview and hypothesis

While preliminary studies have demonstrated the association between mood disorders and lower neurocognitive functioning in Hispanic aging adults, there’s a notable reliance on the Hispanic Community Health Study/Study of Latinos [33] database, which primarily includes data from metropolitan areas with a concentrated Hispanic population [34]. Given that approximately 1.4 million Hispanics reside in rural America, it’s imperative to replicate these findings within this specific demographic [4]. Furthermore, although studies conducted by O’Bryant et al. [7] included rural Hispanic aging adults, they assessed the association of depression and neurocognitive functioning using only one neurocognitive battery (i.e., RBANS). Our study aims to extend this work by assessing the influence of mood disorders on four additional neurocognitive assessments (e.g., CLOXs and TMT) for a comprehensive understanding of neurocognitive functioning scores across domains which has been recommended in prior literature [35]. Moreover, the exploration of anxiety as a separate construct from depression in Hispanic rural aging adults warrants attention. Given the significance of early detection in improving long-term outcomes for neurocognitive functioning, it is critical to identify aging adults who are at risk. Utilizing Myers’ [15] lifespan biopsychosocial model and previous research demonstrating the association between ethnicity and mood disorders on neurocognitive functioning [10, 13, 6, 7], the present study addresses two hypotheses.

#### Hypothesis 1

Higher levels of anxiety and depression will be associated with lower scores on all neurocognitive functioning assessments when controlling for ethnicity, age, income, and gender.

#### Hypothesis 2

Ethnicity will be associated with scores on all neurocognitive functioning assessments when controlling for anxiety (in model 1), depression (in model 2), age, income, and gender. Based on prior studies, we predict Hispanic rural aging adults will score lower than non-Hispanic Whites on all neurocognitive measures.

#### Hypothesis 3

The relationship between ethnicity and neurocognitive functioning will be moderated by depression (model 1) and anxiety (model 2) when controlling for age, income, and gender. Specifically, we predict that the association will vary depending on the levels of depression and anxiety. Also, the relationship will be more pronounced among Hispanic rural aging adults as opposed to non-Hispanic White aging adults.

## METHODS

### Transparency and openness

We report how we determined sample sizes, all data exclusions, all manipulations, and all measures. The data, analysis code, and research materials on which the study conclusions are based can be freely accessed through this Open Science Framework link: https://osf.io/as6vj/. Data were analyzed using R, version 4.3.2 [36]. We did not preregister for this study. The research reported here was approved by the Institutional Review Board at Texas Tech University Health Science Center.

### Participants

Project FRONTIER is an epidemiological study to explore the course of chronic disease development and its longitudinal impact on cognitive, physical, social, and interpersonal functioning in aging among rural adults. Participants were included if they were over the age of 40 (no upper age limit) and living in a rural area in West Texas partnered with Project FRONTIER.

From a sample of 1,864 participants, 752 (40.3%) were non-Hispanic White and 1,053 (56.4%) were Hispanic. The 58 participants who did not identify as non-Hispanic White or Hispanic were eliminated from further analyses, which reduced the total sample size to 1,806 participants. Therefore, only Hispanic rural aging adults and non-Hispanic White rural aging adults were included in the study. Of these, 290 were missing in at least one of the six independent variables used throughout the analyses, which reduced the sample size to 1,516 participants with completed responses on each independent variable. Finally, 54 participants had impossible values on either anxiety, long-term processing and memory, and/or visuospatial/visuoconstructive memory. Removing these participants further reduced the sample size to 1,462 with completed responses on each independent variable. These remaining participants were mostly female (68.9%), Hispanic (55.7%), and averaged 59.4 (*SD* = 12.2) years of age. Of the 814 Hispanic participants, 442 (54.3%) took the test battery in Spanish, while the remainder took it in English. Only two (.003%) of the 648 non-Hispanic White participants took the test battery in Spanish, the remainder took it in English. See Table 1 for a more detailed demographic breakdown.

**Table 1:** Demographic variable breakdown.

**Demographic variable**	***n***
**Gender**	
Male	434
Female	963
Average age (*M*) (*SD*)	59.4 (12.12)
**Ethnicity**	
Hispanic	746
Non-Hispanic White	610
**Language**	
English	912
Spanish	427
**Income**	
<$30,0000	861
>$30,000	536

Discrepancies in cell totals are due to some variables missing small amounts of data.

### Procedure

This study was conducted following IRB approval (Blinded for Review IRB #L06-028) and draws data from Project FRONTIER, an ongoing epidemiological study launched in 2006. Project FRONTIER investigates neurocognitive, biological, and psychosocial outcomes of aging drawn from a defined population residing within rural communities of West Texas, United States. This sampling approach utilized in Project FRONTIER is a strength of the current study, as prior research has demonstrated that the demographic composition of participants recruited from Project FRONTIER closely mirrors the rural population from the counties (i.e., Cochran and Parmer) they were drawn from [37].

Individuals were eligible to participate if they were: 1) aged 40 and above and 2) were enlisted from Cochran, Bailey, Parmer, and Hockley counties of rural West Texas. Project FRONTIER employed a community-based participatory research (CBPR) approach, which actively involves local communities, leveraging relationships with entities like local hospitals, clinics, senior citizen organizations, and community establishments to improve recruitment [37]. Endorsed by the National Institute of Environmental Health Sciences, CBPR is an effective approach to rural health research [38]. Project FRONTIER’s success in participant recruitment evolved by establishing local advisory boards, organizing informative presentations, integrating the local workforce into research operations, and forming partnerships with community organizations to ensure protocol adherence. Recruitment strategies also extend to community events and door-to-door engagements with rural residents.

All participants enrolled in Project FRONTIER met eligibility criteria and voluntarily signed a written consent. Once consented to the study, participants completed a demographic questionnaire (e.g., gender, age, and ethnicity), self-report measures (e.g., anxiety and depression measures), and neuropsychological assessments to index their overall cognitive functioning, rote memory, executive functioning, long-term processing/memory, and visuospatial/visuoconstructive ability. Furthermore, all participants had the option to complete the surveys in either English or Spanish. After participants completed study protocol procedures, they were mailed a feedback letter and copy of their clinical lab results and had the opportunity to designate a health care provider to receive the results as well. All participants who completed the study were compensated $50 for their time.

### Measures

#### Demographic questionnaire

The demographic questionnaire assessed gender, ethnicity (Hispanic vs. non-Hispanic), race, age, income, and preferred language (i.e., Spanish or English).

#### Geriatric depression scale-30

The Geriatric Depression Scale (GDS-30) [39] is a 30-item self-report questionnaire developed to screen for depression in aging adults, independent of physical complaint, focusing on affective and behavioral symptoms of depression. The GDS-30 has been shown to have good score reliability (r = .85) [40], sensitivity, and specificity [41]. Among Hispanic older adults, the GDS-30 yielded good internal consistency values ranging from Cronbach’s *α* of .85 to .88 [42].

#### Beck anxiety inventory

The Beck Anxiety Inventory (BAI) [43] is a self-report measure consisting of 21 items assessing the severity of anxiety symptoms, including somatic and subjective anxiety. The severity of symptoms was rated for each item using a 4-point Likert scale of 0 (Not at all), 1 (Mildly), 2 (Moderately), and 3 (Severely). Thus, the total score ranges from 0–63, with higher scores representing more severe degrees of anxiety symptoms [43]. The BAI has excellent internal consistency, with a mean coefficient alpha of .91 [44] and good test-retest reliability (*r* = .67–.75) [43, 45]. Among Hispanic older adults, the BAI yielded good internal consistency values ranging from Cronbach’s *α* of .83 to .84 [46].

#### Repeatable battery for the assessment of neuropsychological status

The Repeatable Battery for the Assessment of Neuropsychological Status (RBANS) [47] consists of 12 subtests that assess five cognitive domains: Attention (coding and digit span subtests), Language (semantic fluency and picture naming subtests), Visuospatial/Visuoconstructional (line orientation and figure copy subtests), Immediate Memory (list and story memory subtests) and Delayed Recall (list recall and recognition, story recall, and figure recall subtests). The overall score ranges from 40–160, with scores below 78 indicating impaired cognitive functioning [48]. The RBANS demonstrates good reliability and validity [47], as well as good diagnostic accuracy for AD/ADRD [49]. The RBANS has been translated and adapted to Spanish and has yielded sufficient internal consistency with a Cronbach’s *α* of .73 [50]. The RBANS was used in this study as a measure of overall neurocognitive functioning, as prior confirmatory factor analyses found that the RBANS was best suited to assess global cognitive abilities and that the five-factor structure demonstrated poor model fit [51].

#### Clock drawing test

The Clock Drawing Test (CDT) assesses long-term processing, overall memory, and visuospatial/visuoconstructive memory [52]. The CDT has two subtests, CLOX 1 and CLOX 2. The CLOX 1 is a measure of long-term processing and overall memory [53]. Participants are given a blank sheet of paper and are instructed to draw a clock from memory that displays “1:45”. CLOX 2 is a measure of visuospatial/visuoconstructive memory capabilities [53]. Participants observe the examiner drawing a clock and setting the hands to “1:45”, placing the 12, 3, 6, and 9 first and making the hands into arrows. The participant is then instructed to copy the examiner’s clock. Scoring criteria are identical for CLOX 1 and CLOX 2. Total scores range from 0–15, with a score less than 10 reflecting abnormal functioning [54]. One point is given for each accurately drawn aspect of the clock (e.g., circle present, numbers 1–12, correct spacing, minute hand longer than an hour). The clock drawing test is a valid and reliable (Cronbach’s* α* = 0.82) measure to detect neurocognitive impairment in aging adults [55]. The CDT has been translated and adapted to Spanish and has yielded sufficient internal consistency with a Cronbach’s *α* of .82 [56].

#### Trails making test

Overall, the Trails Making Test (TMT) measures rote memory and executive functioning and is an accurate measure in evaluating neurocognitive functioning [57]. The TMT consists of the TMT-A and TMT-B and scores are based on the overall time (in seconds) required to accurately complete the task, with a maximum score of 300 seconds. The TMT was aggregated by scores on TMT-A and TMT-B because confirmatory factor analysis has identified an excellent fit for a two-factor model and a poor fit for a one-factor structure using the composite score [58].

The TMT-A assesses rote memory ability by instructing participants to draw connecting lines to each circle labeled 1–25 in numerically ascending order (i.e., 1-2-3-4-etc.). For individuals aged 55–75, a score of 42 seconds or below is considered normal, with scores above 70 seconds indicating cognitive impairment [59]. For individuals aged 75–98, a score of 51 seconds or below is considered normal, with scores above 79 seconds indicating cognitive impairment [59].

The TMT-B measures executive functioning by instructing participants to connect circles labeled with numbers (1–13) and letters (A-L) in alternating numerical and alphabetical ascending order (i.e., 1-A-2-B-3-C-etc.). For individuals aged 55–75, a score of 101 seconds or below is considered normal, with scores above 273 seconds indicating cognitive impairment [59]. For individuals aged 75–98, a score of 128 seconds or below is considered normal and scores above 273 seconds indicate neurocognitive impairment [59]. TMT has been translated into Spanish and has been normed for Spanish-speaking populations [60]. Two translated versions of TMT have been developed for Spanish speakers, the first version includes the “Ch” sound and the second includes the alphabet “D,” exactly as in the English version [61]. These two versions have been found to produce equivalent results which suggests that the TMT is an acceptable measure to assess executive functioning among native Spanish speakers [61]. It also should be noted that higher scores indicate better neurocognitive functioning on both TMT-A and TMT-B as we took the raw scores and translated them to Z scores utilizing Periáñez and colleague’s [62] norms.

### Analysis plan

All data analyses were conducted in R (Version 4.3.2). Descriptive statistics were conducted for the baseline assessments (see Table 1). Before conducting our analyses, we labeled and dichotomized study variables: gender, ethnicity, and income. Ethnicity was dichotomized into two groups: Hispanic and non-Hispanic White. Income was dichotomized as individuals who made less than $30,000 and those who made $30,000 and up. We used $30,000 as the poverty benchmark in alignment with the U.S. Federal Poverty Guidelines [63]. Gender was dichotomized as male or female. Age, depression, and anxiety remained as continuous variables throughout all inferential analyses. To assess any significant differences between non-Hispanic White and Hispanic rural aging adults on the main study variables at baseline, two ANCOVAs tested the effects of ethnicity on anxiety and depression while controlling for age, income, and gender.

#### Hypothesis 1, 2, and 3

To evaluate our first hypothesis, whether higher levels of anxiety and depression would be associated with lower scores on neurocognitive functioning across five different indices (i.e., overall neurocognitive functioning, rote memory, executive functioning, long-term processing and memory, and visuospatial/visuoconstructive ability), we conducted two separate multivariate analyses of covariance (MANCOVAs). These MANCOVAs also address the second and third hypotheses, which aim to assess whether: a) Hispanic rural aging adults scored lower on neurocognitive functioning and b) whether anxiety and depression potentially moderate the relationship between ethnicity and neurocognitive functioning solely for Hispanic rural aging adults. We decided to conduct MANCOVAs because they allow for the simultaneous examination of these associations in a single analysis [64]. MANCOVAs are also an efficient method to understand how anxiety and depression collectively contribute to multiple neurocognitive domains of functioning while avoiding the potential of inflating Type 1 error [64]. Additionally, literature has supported the use of MANCOVAs when dependent variables are correlated. Given that we are assessing different domains of neurocognitive functioning, which are shown to be highly correlated with each other [65], we decided a MANCOVA for these analyses would provide the most optimal results. For all analyses, significance was assessed at *p* < .05 (two-tailed).

The first MANCOVA explored the combined effects of ethnicity and anxiety, along with their interaction, on the five neurocognitive variables. Age (treated continuously) income (> $30,000 and < $30,000), and gender (male/female) served as covariates. The second MANCOVA explored the combined effects of ethnicity and depression, along with their interaction, on the five neurocognitive variables. Age (treated continuously) income (>$30,000 and <$30,000), and gender (male/female) served as covariates.

After each MANCOVA, we conducted post hoc analyses of covariance (ANCOVAs) to investigate the individual effects of our independent variables on each neurocognitive domain. We report the ANCOVA statistics for each significant main effect and interaction but only interpret the effects that were also significant in the original MANCOVA. To explore the main effects of anxiety and depression, of which are continuous variables, we utilized Pearson correlations to probe the strength and direction of each relationship for each domain of neurocognitive functioning.

## RESULTS

### Missing data

There were 290 participants removed for missing data on any independent variable, so the final sample was 1,462 participants. Independent sample *t*-tests were examined to assess whether the 290 participants who were removed differed significantly from the final 1,462 on any of the eight continuous variables involved in the analyses. Using a Bonferroni corrected alpha value (*α* = .05/8 = .006), *t*-tests revealed that the missing group (*M* = 44.69, *SD* = 17.31) had higher scores on rote memory (*t*(1668) = 4.02, *p* < .001) than the included group (*M* = 39.93, *SD* = 12.57). The missing group (*M* = 53.43 *SD* = 31.86) also had high scores on executive functioning (*t*(1542) = 3.60, *p* < .001) than the included group (*M* = 45.39, *SD* = 17.52). No other significant differences between the missing and included groups on age, depression, anxiety, long-term processing and memory, visuospatial/visuoconstructive memory, or overall cognitive functioning emerged.

Finally, we examined the data for possible differences between a complete case analysis and our current handling of missing data. Eliminating every participant with missing data on any independent or dependent variable eliminates an additional 141 participants, leaving us with 1,321 participants with complete data. However, when these 141 participants are added to the missing group, the missing group significantly differs on six of the eight continuous variables, rather than only two. Therefore, we opted to leave in these 141 additional participants wherever possible to minimize differences between the missing and included groups. Lastly, we want to note that none of the effects differed depending on whether the main analyses were completed with only fully completed cases (i.e., *N* = 1,321) or with those who had complete data on each independent variable (i.e., *N* = 1,462), and therefore, we decided to retain as many participants as possible.

### Ethnic differences in anxiety and depression

An ANCOVA indicated there was no significant difference between Hispanic (*M* = 6.44*, SD* = 5.72) and non-Hispanic White (*M* = 5.08, *SD* = 5.09) rural aging adults on depression scores (*F*(1, 1457) = .50, *p* = .48) controlling for age, income, and gender. However, a second ANCOVA indicated a significant difference between Hispanic (*M* = 4.96*, SD* = 6.72) and non-Hispanic White (*M* = 5.47, *SD* = 6.14) rural aging adults on anxiety scores (*F*(1, 1457) = 18.09, *p* < .001, *η^2^* = .01) controlling for age, income, and gender.

### Hispanic identity and anxiety

The first MANCOVA examined whether anxiety moderated the relationships between ethnicity and the five neurocognitive dependent variables, controlling for age, income, and gender. Results indicated a multivariate effect of ethnicity (*V* = .20, *F*(5,1,310) = 64.69, *p* < .001), a multivariate effect of anxiety (*V* = .02, *F*(5,1310) = 4.57, *p* < .001) when controlling for age, income, and gender on all five indices of neurocognitive functioning. There was no significant interaction effect (*V* < .01, *F*(5,1310) = 1.27, *p* = .28). Because the interaction effect was nonsignificant in the MANCOVA, we did not parse out significant interactions that appeared in the ANCOVAs below. See Table 2 for the complete MANCOVA statistics.

**Table 2:** Effects of ethnicity, anxiety, and covariates on all measures of neurocognitive functioning.

**Effect**	**Pillai’s Trace (*V*)**	***F*(5,1310)**
Ethnicity	0.2	64.69^***^
Anxiety	0.02	4.57^***^
Age	0.07	21.04^***^
Income	0.06	17.04^***^
Gender	0.02	5.60^***^
Ethnicity x Anxiety	0.004	1.27

MANOVA 1 effects of ethnicity with anxiety as a moderator and age, income, and gender as controls, Pillai’s Trace (*V*), test statistics, and *p*-values. ^***^p < .001.

#### Overall cognitive functioning (RBANS)

The ANCOVA for overall cognitive functioning showed a main effect for ethnicity (*F*(1,1,432) = 252.61, *p* < .001, *η^2^* = .15) when controlling for age, income, and gender such that Hispanic rural aging adults (*M* = 78.20, *SD* = 13.25) scored lower on overall cognitive functioning than non-Hispanic White rural aging adults (*M* = 92.22, *SD* = 15.0). There was also a main effect of anxiety (*F*(1,1,432) = 16.12, *p* < .001, *η^2^* = .01, *r* = −.06) when controlling for age, income, and gender such as scores on anxiety increased, scores on overall cognitive functioning decreased. See Table 3 for complete ANCOVA statistics.

**Table 3:** Individual effects in each ANCOVA on each neurocognitive functioning variable.

**Effect**	**Overall cognitive functioning**	**Rote memory**	**Executive functioning**	**Long-term processing/memory**	**Visuospatial/constructive memory**
Ethnicity	252.61^***^	16.98^***^	25.26^***^	49.33^***^	46.36^***^
Anxiety	16.12^**^	2.63	0.05	2.82	2.98
Age	108.44^***^	11.35^***^	2.82	61.25^***^	84.07^***^
Income	92.09^***^	3.22	1.43	17.18^***^	32.45^***^
Gender	25.40^***^	2.86	0.15	17.27^***^	6.80^**^
Ethnicity x Anxiety	1.58	2.1	3.22	3.69	0.92

^**^p < .01, ^***^p < .001; all *df* > 1,1312.

#### Long-term processing and memory (CLOX 1)

The ANCOVA for long-term processing and memory showed a main effect for ethnicity (*F*(1,1,455) = 549.33, *p* < .001, *η^2^* = .03) when controlling for age, income, and gender such that Hispanic rural aging adults (*M* = 11.75, *SD* = 1.92) scored lower on long-term processing and memory than non-Hispanic White rural aging adults (*M* = 12.43, *SD* = 2.09). There was no main effect of anxiety (*F*(1,1,455) = 2.82, *p* = .09). The way interaction between ethnicity and anxiety was not significant (*F*(1,1,455) = 3.69, *p* = .06) when controlling for age, income, and gender.

#### Visuospatial/visuoconstructive memory (CLOX 2)

The ANCOVA for visuospatial/visuoconstructive memory showed a main effect for ethnicity (*F*(1,1,455) = 46.36, *p* < .001, *η^2^* = .03) when controlling for age, income, and gender such that Hispanic rural aging adults (*M* = 13.19, *SD* = 1.40) scored lower on visuospatial/visuoconstructive memory than non-Hispanic White rural aging adults (*M* = 13.69, *SD* = 1.26). There was no main effect of anxiety (*F*(1,1,455) = 2.99, *p* = .08) when controlling for age, income, and gender. The two-way interaction between ethnicity and anxiety was not significant (*F*(1,1,455) = 0.92, *p* = .34) when controlling for age, income, and gender.

#### Rote memory (TMT-A)

The ANCOVA for rote memory showed a main effect for ethnicity (*F*(1,1,431) = 16.98, *p* < .001, *η^2^* = .01) such that Hispanic rural aging adults (*M* = 38.76, *SD* = 13.89) scored lower on rote memory than non-Hispanic White rural aging adults (*M* = 41.35, *SD* = 10.57). There was no effect of anxiety (*F*(1,1,431) = 2.63, *p* = .11). There was no significant interaction effect (*F*(1,1431) = 2.09, *p* = .15) when controlling for age, income, and gender.

#### Executive functioning (TMT-B)

The ANCOVA for executive functioning showed a main effect for ethnicity (*F*(1,1324) = 25.26, *p* < .001, *η^2^* = .02) when controlling for age, income, and gender such that Hispanic rural aging adults (*M* = 42.97, *SD* = 21.27) scored lower on executive functioning than non-Hispanic White aging adults (*M* = 48.07, *SD* = 11.48). There were no other significant main effects or interactions (all *F*s < 3.22, all *p*s > .07) when controlling for age, income, and gender.

### Hispanic identity and depression

The second MANCOVA replicated the first MANCOVA for depression. Results indicated a multivariate effect of ethnicity (*V* = .20, *F*(5,1,310) = 65.80, *p* < .001), a multivariate effect of depression (*V* = .04, *F*(5, 1,310) = 11.38, *p* < .001) when controlling for age, income, and gender on all five indices of neurocognitive functioning. The interaction between depression and age was not significant (*V* = .008, *F*(5,1,310) = 2.16, *p* = .06). See Table 4 for the complete MANCOVA statistics.

**Table 4:** Effects of ethnicity and depression on all measures of neurocognitive functioning.

**Effect**	**Pillai’s Trace (*V*)**	***F*(5,1310)**
Ethnicity	0.2	65.80^***^
Depression	0.04	11.38^***^
Age	0.08	22.74^***^
Income	0.05	14.29^***^
Gender	0.02	5.42^***^
Ethnicity x Depression	0.01	2.16

MANOVA 2 effects of ethnicity with depression as a moderator and age, income, and gender as controls, Pillai’s Trace (*V*), test statistics, and *p*-values. ^***^p < .001.

#### Overall cognitive functioning (RBANS)

The ANCOVA for overall cognitive functioning showed a main effect for ethnicity (*F*(1,1,432) = 210.56, *p* < .001, *η^2^* = .13) when controlling for age, income, and gender such that Hispanic rural aging adults (*M* = 78.20, *SD* = 13.25) scored lower on overall cognitive functioning than non-Hispanic White rural aging adults (*M* = 92.22, *SD* = 15.0). There was also a main effect of depression (*F*(1,1,432) = 48.48, *p* < .001, *r* = −.21), such that as scores on depression increased, scores on overall cognitive functioning decreased. The two-way interaction between ethnicity and age was not significant (*F*(1,1,432) = 3.15, *p* = .08) when controlling for age, income, and gender. See Table 5 for complete ANCOVA statistics.

**Table 5:** Individual effects in each ANCOVA on each neurocognitive functioning variable.

**Effect**	**Overall cognitive functioning**	**Rote memory**	**Executive functioning**	**Long-term processing/memory**	**Visuospatial/constructive memory**
Ethnicity	210.56^***^	18.47^***^	27.58^***^	33.05^***^	41.08^***^
Depression	48.48^***^	5.35^*^	0.56	6.37^*^	22.66^***^
Age	119.58^***^	12.82^**^	3.69	64.14^***^	92.25^***^
Income	75.61^***^	2.12	0.83	14.30^***^	24.05^***^
Gender	25.91^***^	2.62	0.14	17.03^***^	7.30^**^
Ethnicity x Depression	3.15	4.58^*^	5.59^*^	0.83	2.05

Significant interactions were not probed because the interaction terms in the MANOVA were nonsignificant. ^*^p < .05, ^**^p < .01, ^***^p < .001; all *df* > 1,1312.

#### Long-term processing and memory (CLOX 1)

The ANCOVA for long-term processing and memory showed a main effect for ethnicity (*F*(1,1,455) = 33.05, *p* < .001, *η^2^* = .02) when controlling for age, income, and gender, such that Hispanic rural aging adults (*M* = 11.75, *SD* = 1.92) had lower scores on long-term processing and memory than non-Hispanic White rural aging adults (*M* = 12.43, *SD* = 2.09). There was an effect of depression (*F*(1,1,455) = 6.37, *p* = .01, *r* = −.08) when controlling for age, income, and gender, such that as scores on depression increased, scores on long-term processing and memory decreased. The interaction between ethnicity and depression was not significant (*F*(1,1,455) = 0.83, *p* = .36) when controlling for age, income, and gender.

#### Visuospatial/visuoconstructive memory (CLOX 2)

The ANCOVA for visuospatial/visuoconstructive memory showed a main effect for ethnicity (*F*(1,1455) = 41.08, *p* < .001, *η^2^* = .03) when controlling for age, income, and gender such that Hispanic rural aging adults (*M* = 13.19, *SD* = 1.40) scored lower on visuospatial/visuoconstructive memory than non-Hispanic White rural aging adults (*M* = 13.69, *SD* = 1.26). There was an effect of depression (*F*(1,1455) = 22.66, *p* < .001, *r =* −.14) when controlling for age, income, and gender, such that as depression scores increased, visuospatial/visuoconstructive memory scores decreased. There was no interaction between depression and ethnicity (*F*(1,1,455) = 2.05, *p* = .15) when controlling for age, income, and gender.

#### Rote memory (TMT-A)

The ANCOVA for rote memory showed a main effect for ethnicity (*F*(1,1,431) = 18.57, *p* < .001, *η^2^* = .01) when controlling for age, income, and gender, such that Hispanic rural aging adults (*M* = 38.76, *SD* = 13.89) scored lower on rote memory than non-Hispanic White rural aging adults (*M* = 41.35, *SD* = 10.57). There was an effect of depression (*F*(1,1,431) = 5.35, *p* = .02, *r* = −.06) when controlling for age, income, and gender, such that as depression scores, scores on rote memory decreased. The interaction between ethnicity and depression was significant, (*F*(1,1,431) = 4.58, *p* = .03, *η^2^* = .003) when controlling for age, income, and gender but we did not investigate this interaction because it was null in the MANCOVA.

#### Executive functioning (TMT-B)

The ANCOVA for executive functioning showed a main effect for ethnicity (*F*(1,1,324) = 27.58, *p* < .001, *η^2^* = .02) when controlling for age, income, and gender, such that Hispanic rural aging adults (*M* = 42.97, *SD* = 21.27) scores lower on executive functioning than non-Hispanic White rural aging adults (*M* = 48.07, *SD* = 11.48). There were no other significant main effects (all *F*s < 3.69, all *p*s > .06) when controlling for age, income, and gender. The interaction between ethnicity and depression was significant (*F*(1,1324) = 5.59, *p* = .01, *η^2^* = .004) when controlling for age, income, and gender but we do not investigate the interaction because it was nonsignificant in the MANCOVA. See Table 6 for complete group means on each neurocognitive dependent variable.

**Table 6:** Group means for each neurocognitive variable.

**Effect**	**Anxiety**	**Depression**	**Age**	**Ethnicity**		**Income**		**Gender**	
	***Pearson’s r***	***Pearson’s r***	***Pearson’s r***	**Hispanic**	**White**	**<30k**	**>30k**	**Male**	**Female**
Overall Cognitive Functioning	−0.06^*^	−0.21^*^	−0.09^*^	78.20^*^	92.22	79.81^*^	91.86	82.26^*^	85.44
Rote Memory	−0.03	−0.06^*^	−0.05^*^	38.79^*^	41.35	39.03	41.37	39.13	41.3
Executive Functioning	0.02	−0.03	0.01	42.97^*^	48.07	44.21	47.13	45.79	45.21
Long-term Processing/Memory	−0.01	−0.08^*^	−0.14^*^	11.75^*^	12.43	11.77^*^	12.5	11.73^*^	12.19
Visuospatial/Constructive Memory	−0.03	−0.14^*^	−0.17^*^	13.19^*^	13.68	13.17^*^	13.79	13.28^*^	13.47

^*^Indicates significant difference (*p* < .05) from cell to the right or a significant correlation in the anxiety, depression, and age columns.

## DISCUSSION

Depression and anxiety have been linked to impairments in neurocognitive functioning among aging adults [66–68], and previous studies have found that Hispanic individuals score lower on neurocognitive functioning assessments compared to non-Hispanic individuals [7, 8, 10, 12, 37]. This study extends upon the few studies [6, 8] to examine the association between depression, anxiety, and neurocognitive functioning among an underserved and understudied sample of Hispanic and non-Hispanic White rural aging adults.

First, our study examined the differences in scores of anxiety and depression in rural aging adults. There were no significant differences between Hispanic and non-Hispanic rural aging adults on scores of depression. On the other hand, Hispanic individuals reported lower levels of anxiety compared to their non-Hispanic counterparts, but the effect size was marginal with an observed variance of 1%. These results are inconsistent with prior research on this population which demonstrated that Hispanic older adults are 14% more likely to endorse depression symptoms than non-Hispanic older adults [27].

Contrary to our initial hypotheses, depression and anxiety did not moderate the relationship between ethnic identity and neurocognitive functioning—in fact, ethnicity demonstrated a significant and large effect on scores of neurocognitive functioning regardless of the severity of the participant’s depression or anxiety symptoms and when controlling for demographic characteristics (see Table 2). The results of this study are surprising, as an additive effect was expected considering that ethnicity, depression, and anxiety are all significant risk factors for neurocognitive functioning independently. Even after controlling for anxiety and depression, ethnicity emerged as a robust predictor of neurocognitive functioning (i.e., accounting for 20% of the variance in neurocognitive scores) across all five neurocognitive measures, with Hispanic rural aging adults having lower scores on overall neurocognitive functioning compared to their non-Hispanic White counterparts.

Specifically, Hispanic rural aging adults had significantly lower scores on all five indices, which include long-term processing/memory, visuospatial/visuoconstructive ability, rote memory, executive functioning, and overall neurocognitive functioning (see Tables 2 and 4). The findings of the current study support results found in prior studies [14, 56, 69]. For instance, Marquine and colleagues [69] found that Hispanic adults had lower levels of global neurocognitive functioning, executive functioning, learning, recall, working memory, and processing speed compared to non-Hispanic White adults. Another study found that 59% of 1,165 Mexican Americans failed the clock drawing test, indicating poor executive function [56]. The current study extends these findings to a Hispanic rural aging population, which has been underrepresented in research.

Additionally, anxiety exhibited a small and significant effect on overall neurocognitive functioning although it did not significantly predict the other indices of neurocognitive ability, indicating that anxiety might have a broader impact on neurocognitive functioning rather than targeting specific cognitive domains (e.g., executive functioning; see Table 3). Notably, rural aging adults with higher levels of anxiety demonstrated lower scores on overall neurocognitive functioning, suggesting that individuals with anxiety symptoms may be at a greater risk for lower overall neurocognitive functioning. However, given the small effect of anxiety on overall neurocognitive functioning, these results should be interpreted with caution. It is worth stating that anxiety might also influence other aspects of neurocognitive functioning that were not individually examined in this study, such as language, social cognition, and motor skills.

Multiple lines of evidence support the association between anxiety and neurocognitive impairment [70, 71], yet few have examined the association in rural aging adults, aggregated by ethnicity, and less is known about the underlying mechanism for this relationship. One longitudinal study has attempted to establish temporal precedence, hypothesizing that anxiety could be a consequence of cognitive impairment [72], which suggests that anxiety may emerge because of early awareness of neurocognitive impairment. Furthermore, other studies suggest that worry, a specific criterion of anxiety, plays a significant role in driving neurocognitive impairment for those with anxiety [71, 73]. Therefore, future studies should also examine specific item-level responses on anxiety measures and their relationship to neurocognition, as this would provide valuable insights into the nuanced associations between individual symptoms and neurocognitive impairment. Overall, these results demonstrate that anxiety may have a small but significant impact on neurocognitive functioning in aging adults. However, given the scarcity of studies examining this relationship, further research is needed to clarify which neurocognitive domains are impacted by anxiety. This would then allow us to implement early targeted interventions to mitigate the risk of neurocognitive impairment.

In contrast to anxiety, depression exhibited a small and significant effect on multiple domains of neurocognitive functioning, rather than just one domain (see Table 4). More specifically, participants with more depressive symptoms demonstrated lower scores on neurocognitive functioning on three of the five indices assessed (i.e., overall neurocognitive functioning, long-term processing/memory, visuospatial/visuoconstructive memory, and rote memory), with no significant effect observed for executive functioning. These findings suggest that depression may affect multiple domains of cognitive functioning compared to anxiety, which is in line with extensive research supporting the association between depression and neurocognitive impairment [67, 68, 71, 74, 75].

The non-significant association of depression and executive functioning is not surprising, as previous research has yielded equivocal findings regarding executive functioning in depressed aging adults and suggests that executive impairment may vary according to depression type and severity [76, 77; see Table 5]. For instance, Lockwood et al. [76] found a relationship between depression and poor executive functioning, but these findings were observed in a sample of “severely depressed” aging adults with major depressive disorder (MDD), some of whom were taking psychotropic medications, antidepressants, and benzodiazepines during the study. Given that psychotropic medications [78] and benzodiazepines [79] have been found to impact neurocognitive functioning, it is difficult to discern whether executive impairment was due to participants’ prescribed medication or associated with their MDD. Similarly, a systematic review supported the association between depression and executive functioning in six out of eight studies, but the majority of participants (84.5%) were outpatients with MDD [77] and living in urban areas. While it is beyond the scope of this study to distinguish between various types of depression and depression severity, these studies suggest that mild to moderate levels of depression might not significantly impact executive functioning. Therefore, it is plausible that depression may only impair executive functioning when the individuals meet clinical diagnostic criteria.

### Limitations

It is important to interpret these results with certain limitations in mind. First, our study used ethnicity as a proxy for distinct cultural experiences and broader encounters with systems of inequity. The lack of specific indicators for cultural experiences and inequity and grouping of Hispanic populations into one category prevents us from pinpointing which cultural factors contribute to the associations observed between ethnic identity and neurocognitive functioning. In addition, given that all participants were recruited from West Texas and the study utilized CBPR, our study results may not be representative of other Hispanic aging adults in the United States since participants were not recruited from a known population. We also did not distinguish between those born in the United States and those born in their country of origin. As a result, we were unable to capture the diverse cultural, linguistic, and historical differences present in Hispanic communities that span 20 countries, as recommended by Lopez et al. [9]. Supporting this recommendation, prior studies have found differences in the risk of neurocognitive impairment between individuals of Mexican [80] and Caribbean background [81].

Another limitation of the current study was the inability to adjust income for inflation over time. Without adjusting for inflation, income figures from different periods may not be directly comparable; however, income was used as a control variable. Education and occupation were also not controlled for in the study and may have influenced the results. However, a meta-analysis and systematic review of longitudinal studies found poor and inconsistent evidence to support the association between education and cognitive performance [82], which suggests that the exclusion of education in the current study’s analyses may not have severely influenced study results. Lastly, it is important to note that this study is cross-sectional in nature, which restricts our ability to draw causal inferences.

### Clinical implications and future directions

The present study suggests that Hispanic rural aging adults tend to have lower scores on neurocognitive functioning measures compared to non-Hispanic White rural aging adults. Still, future research is needed to understand the relationships found in this study. The underlying mechanism of why this difference exists is unknown. Therefore, prospective researchers may consider exploring how environmental (e.g., poverty), social (e.g., discrimination; acculturative stress), and structural (e.g., access to healthcare) inequities impact neurocognitive functioning as opposed to using ethnicity to represent the likelihood of lifetime cumulative disadvantages. Furthermore, considering the cultural, linguistic, and historical diversity of Hispanic communities [9], aggregating research results of Hispanic populations by country of origin might provide more nuanced evaluations of neurocognitive risk not captured in this study. Furthermore, while both anxiety and depression were significantly associated with neurocognitive functioning, researchers may consider examining how item-level responses, severity levels, and subtypes of anxiety and depression may influence neurocognitive functioning. This would improve early identification of persons with lower neurocognitive functioning and improve the ability to treat these individuals.

Findings from the current study highlight the potential impact of mood disorders on neurocognitive functioning in rural aging adults. The results also point to the egregious neurocognitive disparities apparent between Hispanic and non-Hispanic White aging rural adults. Mental health interventions that target depression and anxiety for rural aging adults might serve to mitigate potential neurocognitive impairments. Moreover, this study calls attention to the public health need to provide an interdisciplinary approach to supporting Hispanic rural aging adults. Given the links between anxiety, depression, and neurocognitive functioning in this population, along with the limited availability of mental health specialists [83], primary care providers—who are trusted figures within rural communities [84]—can play a crucial role in facilitating access to mental health support for aging adults. Such support not only addresses mental health concerns but also has the potential to mitigate the progression of neurocognitive impairment. Culturally appropriate interventions for rural-dwelling aging adults are warranted, which include utilizing unique platforms (e.g., telehealth) for care. One method is to include rural community stakeholders in the development of mental health interventions and research to ensure studies are built for them and informed by them. Community-based interventions have been shown to be effective and culturally responsive in addressing disparities and improving health outcomes in racial-ethnic minorities [85, 86]. Given the entrenched health disparities in neurocognitive and mental health, it is essential that research enhances our understanding of these associations to support the cognitive health of at-risk Hispanic rural aging adults both in research and in practice.
